# Foliar Application of Nanoclay Promotes Potato (*Solanum tuberosum* L.) Growth and Induces Systemic Resistance against Potato Virus Y

**DOI:** 10.3390/v14102151

**Published:** 2022-09-29

**Authors:** Dalia G. Aseel, Ahmed Abdelkhalek, Fatimah O. Alotibi, Marwa A. Samy, Abdulaziz A. Al-Askar, Amr A. Arishi, Elsayed E. Hafez

**Affiliations:** 1 Plant Protection and Biomolecular Diagnosis Department, Arid Lands Cultivation Research Institute, City of Scientific Research and Technological Applications, New Borg El Arab City 21934, Egypt; 2 Department of Botany and Microbiology, College of Science, King Saud University, P.O. Box 2455, Riyadh 11451, Saudi Arabia; 3 School of Molecular Sciences, The University of Western Australia, Perth, WA 6009, Australia

**Keywords:** nanoclay, potato, PVY, FTIR, SEM, TEM, antioxidant enzymes, gene expressions

## Abstract

Potato virus Y (PVY) is one of the most harmful phytopathogens. It causes big problems for potatoes and other important crops around the world. Nanoclays have been extensively studied for various biomedical applications. However, reports on their interactions with phytopathogens, particularly viral infections, are still limited. In this study, the protective activity of Egyptian nanoclay (CE) and standard nanoclay (CS) against PVY was evaluated on potato (*Solanum tuberosum* L.) plants. Their physicochemical and morphological properties were examined with scanning electron microscopy (SEM), transmission electron microscopy (TEM), Fourier-transform infrared spectroscopy (FTIR), and energy dispersive spectrometer (EDS). SEM and TEM analyses revealed that CE has a spherical and hexagonal structure ranging from 20 to 80 nm in size, while CS has boulder-like and tubular structures of about 320 nm in size. FTIR and EDS showed that both nanoclay types have different functional groups and contain many vital plant nutrients that are necessary for every stage and process of the plant, including development, productivity, and metabolism. Under greenhouse conditions, a 1% nanoclay foliar application enhanced potato growth, reduced disease symptoms, and reduced PVY accumulation levels compared with non-treated plants. Significant increases in levels of antioxidant enzymes (PPO and POX) and considerable decreases in oxidative stress markers (MDA and H_2_O_2_) were also reported. Moreover, a significant increase in the transcriptional levels of defense-related genes (*PAL-1*, *PR-5*, and *CHI-2*) was observed. All experiment and analysis results indicate that the CE type is more effective than the CS type against PVY infection. Based on these results, the foliar applications of nanoclay could be used to manage plant viral infections in a way that is both effective and environmentally friendly. To our knowledge, this is the first report of the antiviral activity of the foliar application of nanoclay against PVY infection.

## 1. Introduction

The potato (*Solanum tuberosum* L.) is a substantial and economical crop. Plant diseases are brought about by parasitic infections caused by oomycetes, microbes, viruses, parasitic plants, nematodes, or organisms and cause significant damage [1,2]. Plant phytopathogens are responsible for 20 to 40% of annual output losses [3]. Furthermore, potato viruses usually damage the economy, hurting both the quality of the seeds and trade [4]. *Potato virus Y* (PVY) is potatoes’ major aphid-transmitted viral pathogen worldwide. It could impact the production of certified seeds and crops grown for processing or fresh markets [5]. PVY (family *Potyviridae* and genus *Potyvirus* of the largest group of plant viruses) has 111 recognized and 86 tentative species infecting over 30 plant families [6]. The different strains of PVY are closely connected with variable levels of pathogenicity, with recombinant being the most important and prevalent [7]. PVY^O^ is a popular strain that causes mild mosaic, mottle, bronzing, and rugosity when the infection is mixed with PVA, PVX, and PVS [8]. Leaf yellowing or necrosis, leaf dropping, and sometimes premature death are the primary symptoms of PVY.

Protecting food crops from viral infections is an important agricultural concern. Traditional ways to stop viral diseases include killing vectors with insecticides, using natural predators, and putting up physical barriers such as reflective mulches and UV-absorbing coverings [9]. Diseases have also been stopped by removing weeds, using virus-free materials, planting seeds early, giving crops time to rest, and throwing away diseased crops [10,11]. Creating crop varieties resistant to a disease is a good and sustainable approach to integrated agriculture [12]. However, creating varieties with the proper spectrum of resistance may require considerable effort and resources [13]. In some cases, breeding cultivars with higher levels of resistance is a good way to reduce crop loss caused by viruses [14]. The use of attenuated virus strains to boost the resistance responses is another possibility [15]. Furthermore, organic farming entails cultivating plants without synthetic fertilizers, antibiotics, pesticides, etc., that could be applied for sustainable agriculture [16].

In recent years, nanoparticles have been attracting increasing attention in agriculture due to their wide range of interesting biological activities, including medical, environmental, and industrial types [17,18]. The use of nanoparticles as a pesticide has become commonplace in recent years to combat a wide variety of phytopathogens [19]. However, their efficacy against viral infections is restricted. Recently, clay, a natural mineral, has been used to fabricate nanocomposites. Clay is a good choice for making high-performance composite materials because it is cheap and easy to find in nature. Naturally occurring biological nanoparticles (such as nanoclay, viruses, tomato carotenoid lycopene, lipoproteins, many chemicals derived from soil organic matter, exosomes, ferritin, and magnetosomes) have a variety of structures and biological functions. Biological nanoparticles are frequently biocompatible and have a repeatable structure [20]. Clay is a fine material created from natural rocks or soil that contains many minerals, a small number of metal oxides, and organic content. Clay is composed of small crystallites of alumino-silicates in different amounts, with iron and magnesium replaced by alkalis and alkaline earth elements [21].

The induction of pathogenesis-related (PR) proteins in plants is one of the most critical defense responses [22]. However, as far as we know, none of them have been linked to virus infection inhibition. Still, PR protein induction is a key part of SAR. These proteins show how this process and the signaling processes that lead to SAR and PR protein accumulation are happening. The thaumatin-like protein gene (*PR-5*) is considered to be an essential antimicrobial weapon. It improves resistance to a wide range of pathogens when overexpressed in tobacco or wheat plants [23,24]. The first step in the main phenylpropanoid pathway is the regulation of the phenylalanine ammonia-lyase 1 gene (*PAL-1*) gene, which serves as the starting point for the biosynthesis of a variety of important compounds such as lignans, coumarins, and flavonoids [24]. Additionally, *PAL* is used in many plants as a marker for induced resistance. This means that *PAL* expression could be changed to make plants more resistant to disease [25]. Additionally, the chalcone isomerase 2 gene (*CHI-2*) has a defensive role in plant immunity by regulating the pathogen-dependent accumulation of isoflavonoid phytoalexins and flavonoids [26].

The main objective of the current work was to use Egyptian nanoclay and standard nanoclay as anti-viruses. These two types of nanoclay are not commercially used in plant disease control, but both are used for industrial purposes, especially in oil mining. The other objectives of the current work were to characterize Egyptian nanoclay and standard nanoclay with FTIR (Fourier-transform infrared spectroscopy), SEM (scanning electron microscopy), TEM (transmission electron microscopy), and EDS (energy dispersive X-ray spectrometer). The effects of foliar nanoclay applications on virus symptoms, plant growth, and the accumulation of PVY inside infested tissues were evaluated for the first time. The activities of reactive oxygen species exploring two enzymes (POX and PPO) and non-enzymatic oxidative stress markers (MDA and H_2_O_2_) were estimated. Moreover, the effect of the foliar nanoclay application on potatoes was shown to induce an immune response against PVY using β-1,3-glucanases (*PR-2*), thaumatin-like protein (*PR-5*), phenylalanine ammonia-lyase-1 (*PAL-1*), and chalcone isomerase 2 (*CHI-2*). Using the foliar spraying of nanoclay as a treatment against PVY infection could be crucial to establishing effective viral disease control strategies. 

## 2. Materials and Methods

### 2.1. Plant Materials and Source of the Viral Isolate

Virus-free potato (*Solanum tuberosum* L.) tubers of the Spunta cultivar were kindly provided by the International Potato Center at the Ministry of Agriculture and Land Reclamation, Egypt. The PVY strain (PVY-Borg El-Arab, Alexandria, Egypt) used in this study was previously isolated from infected potato plants [27] and continuously maintained on *Nicotiana glutinosa* plants for virus propagation under greenhouse conditions.

### 2.2. Source of Nanoclay

Egyptian alluvial soil samples were randomly collected from the top 15 cm of depth of a field, air-dried, grinded, and sieved at <2 mm. The soil sample was pretreated with distilled water, sodium acetate (0.5 M), and 30% hydrogen peroxide to remove salts, carbonates, and organic debris [28]. The soil was thoroughly rinsed with distilled water to completely remove any remaining hydrogen peroxide and sodium acetate residues. In 2 L cylinders filled with distilled water, the prepared soil material was mechanically disseminated and broken up before being let to stand for 24 h to allow for sedimentation. The fine fraction (<2 μm) was syphoned off and dried for 72 h at 40 °C in an oven. Following grinding, the collected clay particles were sieved through a 250 μm sieve and kept in a plastic container for later use. A ball mill was used to mechanically synthesize tiny clay particles (E-max Retch). The milling process was carried out for three hours at a disc speed of 700 rpm [29]. On the other hand, the standard nanoclay was bought from Southern Clay Products, Inc., Gonzales, TX, USA. 

### 2.3. Nanoclays Characterization

#### 2.3.1. SEM

The morphological structure properties of the Egyptian and standard nanoclay were characterized with SEM (JSM-6360 LA, JEOL, Akishima, Tokyo, Japan) at an accelerating voltage of 10 kV. Briefly, a sample of nanoclay was stacked over a holder. Then, it was gold-sputtered so that it could be examined. The sample was scanned to find out how it was put together and to estimate the size of the particles at different magnification levels. 

#### 2.3.2. FTIR

Fourier-transform infrared (FTIR) spectroscopy was performed in transmission mode over the wave number range of 4000−500 cm^−1^ with a Nicolet (Madison, WI, USA) FTIR impact 410 spectrophotometer using KBr pellets. The samples were made by maintaining a ratio of approximately 1:20 between finely ground composite materials and KBr. In a mortar, the powder sample and KBr were ground to a uniform consistency. The pellets were created by compressing a 5 mg sample mixture in a hydraulic press.

#### 2.3.3. TEM

The TEM technique was applied to confirm that the Egyptian and standard nanoclay was within the nanoscale range [30]. Briefly, 20 μL of an aqueous suspension of nanoclay was applied to the carbon-coated side of a grid. After one minute, the slide was cleaned with 10 to 15 drops of distilled water, stained with 1% uranyl acetate for appropriate contrast, placed on grids, and analyzed. TEM images of dried grids were analyzed with a JEOL JEM-2100 (Akishima, Tokyo, Japan), operating at 120 kV.

#### 2.3.4. EDS

Egyptian and standard nanoclay composition analysis was performed with a TEM (JEM-2100, Akishima, Tokyo, Japan) attached to an EDS spectrometer [31]. In brief, nanoclay samples were deposited on a carbon-coated copper grid. High-resolution-TEM (HR-TEM) imaging and EDS elemental analysis were performed on a JEOL-2100F TEM (cold-field-emission gun, 200 kV). Thermo Scientific Pathfinder X-ray Microanalysis Software (Thermo Fischer Scientific, Waltham, MA, USA) was used to process and analyze EDS data. The EDS detector, which has a resolution of 127 eV and a spot size range of 0.5–2.4 nm, was used to conduct the elemental chemical analysis of certain sites of the HR-TEM images. 

### 2.4. Greenhouse Experimental Design

Potato tubers were grown in plastic pots (30 cm in diameter) filled with 4 kg of sterilized soil containing an equal mix of clay and sand (1:1). The experiment was divided into six treatments, each with five replicate pots and three potato tuber/pot. After 30 days of growth, each potato plant’s two true upper leaves were dusted with carborundum (600 meshes) and mechanically inoculated with a freshly prepared PVY inoculum using the forefinger method. The PVY inoculum was prepared by pulverizing infected *N. glutinosa* tissue at a ratio of 1:10 (W/V) in a 100 mM sodium phosphate buffer, pH 7.0, containing 0.5% 2-mercaptoethanol. The first treatment (control (C) treatment) comprised the plants dusted with carborundum and inoculated with free-virus inoculation buffer. The second treatment (virus (V) treatment) comprised plants only inoculated with PVY. The third treatment (CE) comprised plants dusted with carborundum and foliar-sprayed with Egyptian nanoclay. The fourth treatment (CE and V) contained plant foliar spraying with just Egyptian nanoclay 48 h before PVY inoculation. The fifth treatment (CS) comprised plants dusted with carborundum and only foliar-sprayed with standard nanoclay. The sixth treatment (CS and V) comprised plants that were only foliar-sprayed with standard nanoclay 48 h before PVY inoculation. The foliar nanoclay solution was a 1% (*w*/*v*) suspension in sterile distilled water. With a handheld pressure sprayer, whole plant shoots were foliar-sprayed until runoff and the nanoclay solution appeared to cover the leaves. All pots were kept in a greenhouse at 28 °C/16 °C day and night with a relative humidity of 65% and observed daily for the recording of symptom development. At 28 days after PVY inoculation (dpi), potato leaves (3 leaves/plant) of five biological replicates of each treatment were collected and kept at −80 °C until use. In addition, plants were carefully removed, cleaned under running water, and evaluated for their height, shoot and root lengths, and shoot and root fresh weight across all treatments.

### 2.5. Determination of Oxidative Stress Markers

#### 2.5.1. Malondialdehyde (MDA)

Following the work of Heath and Packer [32], all treatments measured MDA levels with thiobarbituric acid (TBA). In brief, 100 mg of tomato leaf samples was crushed in 1 mL of 0.1% trichloroacetic acid (TCA) and centrifuged at 10,000 revolutions per minute for 30 min. One milliliter of sample supernatants was combined with 4 mL of a TBA solution (0.5% TBA: 20% TCA) and incubated at 95 °C for 30 min. Immersion in ice halted the process, and the generated color was measured at 600 nm to determine the MDA concentration (µM/g of fresh weight).

#### 2.5.2. Hydrogen Peroxide (H_2_O_2_)

The fresh plant samples were analyzed for H_2_O_2_ using KI, as described by Junglee et al. [33], with a few modifications. First, 100 mg of fresh plant samples was homogenized in 0.1% TCA and centrifuged to obtain a transparent homogenate. By mixing 1 mL of plant homogenate with 2 mL of a KI solution, the H_2_O_2_ reaction was carried out (1 M KI in 10 mM phosphate buffer, pH 7.0). After 20 min, the absorbance of the reaction was measured at 390 nm using the H_2_O_2_ extinction coefficient (0.28 M^−1^ cm^−1^), and the findings are reported as µM/g fresh weight.

### 2.6. Evaluation of Antioxidant Enzymes Activity 

#### 2.6.1. Peroxidase (POX)

The evaluation of POX activity was conducted in accordance with Angelini et al. [34]. To conduct the test, 500 µL of 5 mM guaiacol and 120 µL of hydrogen peroxide were mixed with 1200 µL of a 100 mM phosphate buffer and 80 µL of a plant extract. After heating the mixture for 10 min at 30 °C, the absorbance at 480 nanometers was measured. This measurement was utilized to derive findings using an extinction coefficient of Ɛ = 26,600 M^−1^ cm^−1^.

#### 2.6.2. Polyphenol Oxidase (PPO)

The activity of PPO was determined using the quinone method [35]. In brief, 500 µL of a crude plant extract was mixed with 1 mL of 50 mM quinone (in 100 mM Tris-HCl buffer pH 6.0) and incubated at 25 °C for 10 min. The absorbance of the reaction was measured at 420 nm, where a 0.001 increase in absorbance equaled one unit of enzyme activity/min and is expressed as µM/g fresh weight.

### 2.7. Analysis of Defense-Related Gene Expression Levels

#### 2.7.1. Extraction of Total RNA and cDNA Synthesis

At 28 days post-inoculation, 100 mg of fresh-weight potato leaves was harvested and subjected to total RNA extraction using the RNeasy Plant Mini Kit (QIAGEN, Hilden, Germany). Following the quality control of the isolated RNA, 2 mg of DNase-treated RNA was utilized to synthesize cDNA in a reverse transcription reaction (M-MuLV Reverse Transcriptase, Biolabs, New England), as described in our earlier investigations [36]. The reverse transcriptase process was carried out at 40 °C for 1 h and deactivated at 80 °C for 10 min in a thermal cycler (Eppendorf, Hamburg, Germany). We placed the reaction mixture in the freezer at −20 °C until we were ready to utilize it.

#### 2.7.2. Quantitative Real-Time PCR (qPCR)Assay and Data Analysis

Using qPCR, the effects of nanoclay on the accumulation of PVY defense-related genes were analyzed. This investigation utilized a distinct set of *PR-2*, *PR-5*, *PAL-1*, *CHI-2*, and *PVY-CP*-specific primers (Table 1). The housekeeping gene-actin was the reference gene utilized to standardize the transcript expression levels (Table 1). The reactions for each sample were conducted in triplicate using the Rotor-Gene 6000 QIAGEN (ABI System, Hilden, Germany) and the SYBR^®^ Green RT Mix (Bioline, Luckenwalde, Germany) [37]. As previously described [38], the target gene’s amplification program and relative expression level were properly quantified and calculated.

### 2.8. Statistical Analysis

The analyzed data were statistically evaluated using one-way ANOVA using the CoStat software (version 6.311, CoHort, Monterey, CA, USA). At the same time, Tukey’s honest significant differences method (HSD) was used to determine statistical differences in the mean at a *p ≤ 0.05* level of probability, and the standard deviation (±SD) is displayed as a column bar. Columns with the same letter do not significantly differ.

## 3. Results and Discussion

### 3.1. Nanoclay Characterization

#### 3.1.1. Scanning Electron Microscopy Analysis

**Figure 1:**
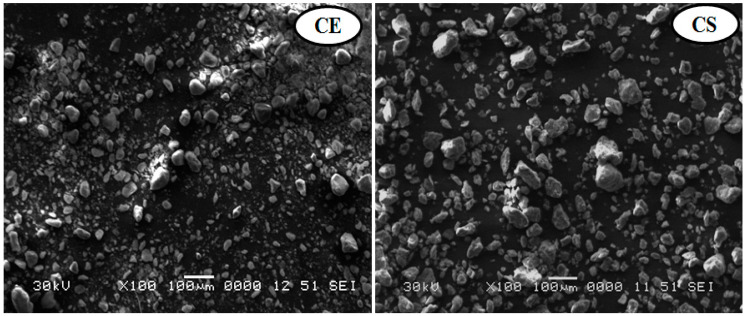
SEM images of Egyptian nanoclay (CE) at 0.2 µm and standard nanoclay (CS) at 0.5 µm.

SEM is a powerful method for examining surface morphology with the direct visualization of nanoparticles [39]. In the current study, SEM analysis revealed that the morphological characteristics showed boulder-like spherical and irregular particles for the Egyptian and standard nanoclays (Figure 1). These results were similar to those shown in previous SEM micrographs that revealed the surface morphologies of nanoclay had changed due to phosphatase adsorption, resulting in the formation of boulder-like structures and rough surfaces [30]. Furthermore, when pure aluminum, Al-2 percent nanoclay, and Al-10 percent nanoclay powders were mixed and compared, it was found that the size and number of aluminum particles decreased as the amount of nanoclay particles increased [40].

#### 3.1.2. Transmission Electron Microscopy Analysis

**Figure 2:**
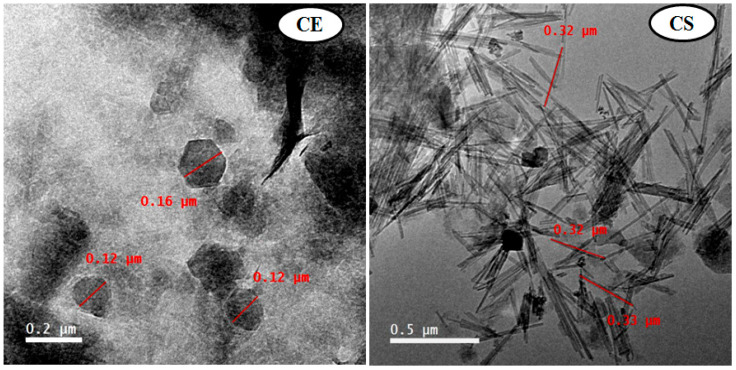
TEM images of Egyptian nanoclay (CE) at 0.2 µm and standard nanoclay (CS) at 0.5 µm.

TEM images of both the nanoclays used in this study are shown in Figure 2. The Egyptian nanoclay powder was observed to have a hexagonal structure with a side distance of 20−80 nm, as shown in Figure 2. The standard nanoclay powder showed tubular structures of about 320 nm in size, as shown in Figure 2. The dark regions in the TEM micrographs occurred due to the overlapping clay mass. Similarly, the nanoclay particles were found to have the hexagonal shape of kaolinite nanoclay crystals converted to bar formations under TEM images. Additionally, intercalation was confirmed by the shrinkage of kaolinite particles and the delamination of kaolinite booklets. The overlapping clay mass caused the dark spots in the TEM photographs [41]. 

#### 3.1.3. FTIR Analysis

**Figure 3:**
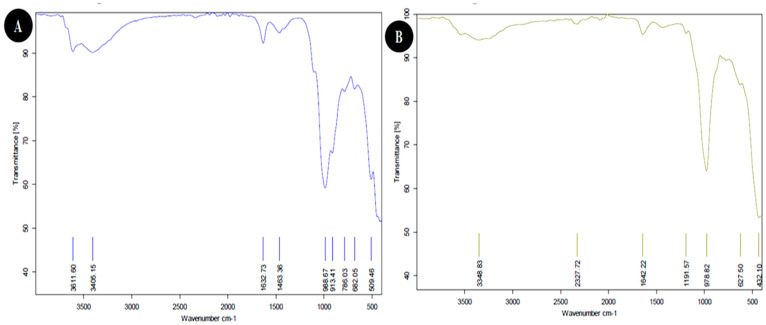
FTIR spectra of Egyptian nanoclay (**A**) and standard nanoclay (**B**).

FTIR spectroscopy revealed the functional groups in both nanoclay. The FTIR spectrum of Egyptian nanoclay showed several peaks (Figure 3A). Data in Table 2 show a broad peak at 3611.60 cm^−1^ that indicates the presence of hydrogen-bonded groups and could correspond to the (O-H) stretching of inner-surface hydroxyl groups. In comparison, the peak at 3405.15 cm^−1^ represents OH. The band at 1632.73 cm-1 indicates H-O-H deformation, and the peak at 1463.36 cm^−1^ represents C–H stretching. The band at 988.67 cm^−1^ suggests OH deformation linked to 2Al^2^; the peak at 913.41 cm-1 can be assigned to C=C binding (alkane); and the bands at 786.03 cm^−1^, 682.05 cm^−1^, and 509.46 cm-1 represent Si-O Quartz, Si-O-Si bending, and Fe-O Fe_2_O_3_ Si-O-Al stretching, respectively. Likewise, the FTIR spectrum of the standard nanoclay showed two different peaks, as shown in Figure 3B that were not present in the Egyptian nanoclay (as shown in Table 2), i.e., the peak at 2327.72 cm^−1^ representing O=C=O carbon dioxide (carbonyl bond group) and the band at 1191.57 cm^−1^ assigned to Al-O as an Si cage (TO^4^). These results support previous reports that indicated that the broadband at 3432 cm^−1^ is due to the stretching vibration of OH groups in the structure of allophane and imogolite [41,42,43,44]. The broadband near 3432 cm^−1^ can be attributed to the stretching vibration of OH groups, and the bands at 1090–940 cm^−1^ can be attributed to the Si–O stretching vibration of orthosilicate anions and Si–O–Al groups [42,43,45,46,47,48]. Allophane has bands ranging from 670 to 430 cm^−1^ [43], whereas imogolite has bands at 500, 420, and 350 cm^−1^ [45]. Consequently, we suggest that some functional and active groups work as viral triggers and prevent the virus’ movement from cell to cell, and the rest can aid viral enzymatic hydrolysis. Furthermore, the nanoclay particles used in anti-virus activity against PVY were found to have a positive effect in terms of anti-oxidation improvement in the potato plants.

#### 3.1.4. EDS Analysis

**Figure 4:**
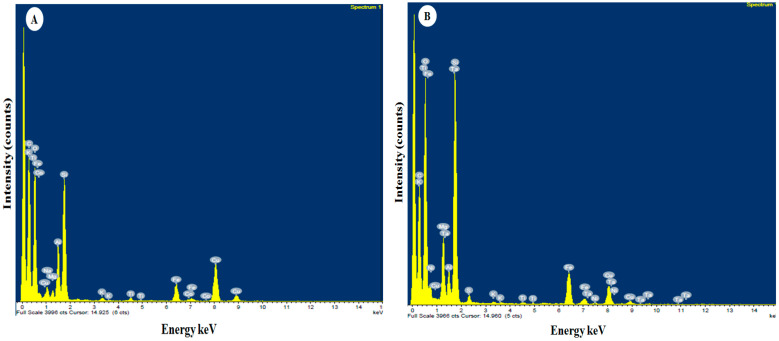
EDS analysis of Egyptian nanoclay (**A**) and standard nanoclay (**B**).

The energy dispersive spectrometer (EDS) spectrum and quantitative analysis of Egyptian nanoclay confirm the presence of elements such as carbon (C), oxygen (O), sodium (Na), magnesium (Mg), aluminum (Al), silicon (Si), potassium (K), titanium (Ti), iron (Fe), cobalt (Co), and copper (Cu), as shown in Figure 4A and Table 3. On the other hand, the EDS spectrum of the standard nanoclay confirmed the presence of carbon, oxygen, magnesium, aluminum, silicon, sulfur (S), potassium, titanium, iron, nickel (Ni), copper, and tantalum (Ta), as presented in Figure 4B and Table 3. We used EDS to determine and distribute the elements for both nanoclays. Our results were similar to those of [48], who reported that the constituent elements and frequency distribution of the elements in their nanocomposite were identified using EDS and EDS-MAP analysis. The presence of O, Si, and Ce in the new nanocomposite was confirmed with EDS analysis. The nanocomposite’s O, Si, and Ce atomic weights were determined. EDS-MAP was used to quantitatively observe the distribution and dispersion the O, Si, and Ce in a specific area of the nanocomposite. In similar experiments for generating Al composites, EDS revealed no chemical reaction between the Al matrix and reinforcement, even though the interfacial connection between them was strong enough [49]. In addition, plant nutrients are necessary for every stage and process of a plant, including emergence, development, productivity, metabolism, promotion, and protection. By improving plant resistance and managing mineral nutritional status, several of these minerals may even be used to protect agricultural plants from both abiotic and biotic stressors [50].

### 3.2. Plant Growth Evaluation

**Figure 5:**
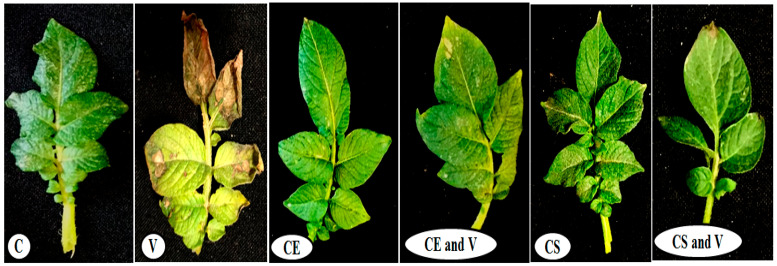
Effect of the foliar application of nanoclays on PVY on potato plants under greenhouse conditions. C: control potato plants; V: potato plants inoculated with PVY; CE: potato plants treated with Egyptian nanoclay; CE and V: potato plants inoculated with Egyptian nanoclay 48 h before PVY inoculation; CS: potato plants treated with standard nanoclay; CS and V: potato plants inoculated with standard nanoclay 48 h before PVY inoculation.

Under greenhouse conditions, the effects of both nanoclays on PVY symptom development, as well as their protective activity against PVY, were assessed. The results showed that the potato plants sprayed with 1% nanoclay 48 h before PVY inoculation had enhanced plant growth and decreased disease severity compared with non-treated plants (Figure 5). At 28 dpi, the PVY-infected potato plants (V treatment) showed leaf drop, necrotic lesions, yellow flecking, and size decreases of some plant leaves (Figure 5), similar to previously reported results [27]. No symptoms were observed on other treatments (C, CE, CS, CE and V, and CS and V) at 28 dpi (Figure 5). For plant growth evaluation, the features of the shoot and root systems were significantly affected by the nanoclay applications compared with the control and PVY treatments (Table 4). The CE treatment showed the highest plant height (37.66 cm), followed by CS (35.66 cm), CE and V (30.33 cm), and CS and V (29.83 cm). On the other hand, a significant reduction was observed in the virus treatment (14.50 cm) compared with the control (24.33 cm). Moreover, root length increased more with the CE (22.66 cm) and CE and V (13.66 cm) treatments than shoot length for the same treatments. Treatment with Egyptian nanoclay significantly increased the fresh weights of potato plant shoot systems (5.27 g) and root systems (0.93 g). There was also a significant increase in fresh weight for shoots and root systems after treatment with Egyptian nanoclay before PVY inoculation (3.77 g and 0.78 g, respectively). In contrast, significant reductions in the fresh weights of the shoot and root systems of potato plants only infected with PVY were observed (1.57 g and 0.5 g, respectively). Likewise, the standard nanoclay treatment was observed to have a highly significant effect on plant height (35.66 cm) compared with treatment with standard nanoclay before PVY inoculation (29.83 cm). Highly significant increases were observed for the shoot and fresh root weight in treatment with only standard nanoclay (4.67 g and 1.16 g, respectively). In comparison, the shoot and fresh root weight in treatment with standard nanoclay 48 h before PVY inoculation significantly decreased (2.23 g and 0.81 g, respectively) compared with the potato plant control (Table 4). In this context, several previous reports have shown that the foliar application of nanoparticles significantly enhanced plant growth, decreased disease severity, and decreased the accumulation levels of many plant viruses inside plant tissues [51,52,53]. 

### 3.3. Effect of Nanoclays on Oxidative Stress Markers

**Figure 6:**
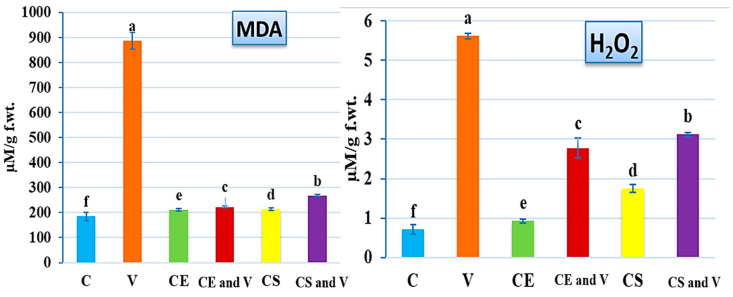
Effect of the foliar application of nanoclay on the MDA and H_2_O_2_ of potato plants at 28 days after PVY inoculation. C: control potato plants; V: potato plants inoculated with PVY; CE: potato plants treated with Egyptian nanoclay; CE and V: potato plants inoculated with Egyptian nanoclay 48 h before PVY inoculation; CS: potato plants treated with standard nanoclay; CS and V: potato plants inoculated with standard nanoclay 48 h before PVY inoculation. The columns reflect the average value of five biological replicates, while the bars represent the standard deviation (SD). According to Tukey’s HSD test (*p* ≤ 0.05), the values of each column following the same letter (a/b/c/d/e/f) do not significantly differ.

In response to several biotic and abiotic stressors, plants produce ROS, including hydrogen peroxide and superoxide, which can either improve stress adaption in certain situations or mediate symptom development in others [54]. Since increased levels of reactive oxygen species (ROS) are characteristic of a viral plant infection [55], measuring ROS is directly correlated with the severity of an infection. For this reason, two oxidative stress indicators (MDA and H_2_O_2_) were measured in all treatment groups (Figure 6). The results showed that the PVY infection (V treatment) increased the levels of MDA (886.61 ± 33.78 µM/g f.wt.) and H_2_O_2_ (5.61 ± 0.09 µM/g f.wt.) in virally infected tissues by about 381% and 690%, respectively, compared with control plants (184.48 ± 15.87 and 0.71 ± 0.12 µM/g f.wt. for MDA and H_2_O_2_, respectively) (Figure 6). These results are consistent with those found in many other studies of viral infections in plants [56,57,58,59,60]. The increase in the level of oxidative stress markers in plant cells that are infected is thought to be a defense mechanism against infection. However, the unbalanced levels of ROS lead to the oxidation of vital cell components such as protein, DNA, and unsaturated fatty acids, which causes plant cells to deteriorate and ultimately leads to infection [61,62]. The treatment of tomato plants with the two types of nanoclay revealed significant reductions in the two stress markers, with an advantage by Egyptian nanoclay (Figure 6). The CE and V treatment led to higher reductions in MDA (222.23 ± 4.50 µM/g f.wt.) and H_2_O_2_ (2.77 ± 0.25 µM/g f.wt.) levels than that reported in the CS and V treatment, with 267.46 ± 4.22 and 3.12 ± 0.05 µM/g f.wt. for MDA and H_2_O_2_, respectively (Figure 6). These results suggest that applying nanoclay to leaves, especially the Egyptian type, is a good way to reduce oxidative stress and lipid peroxidation in virus-infected plants. It was reported that the signs of oxidative stress might be linked to the decreased activity of both enzymatic and non-enzymatic antioxidants, as well as proline and total phenolic compounds [63]. Moreover, the phytohormones have a significant role in controlling plant–potyvirus and plant–insect interactions. For instance, plants can utilize ethylene (ET), jasmonic acid (JA), and salicylic acid (SA) to fight against a variety of phloem-feeding insects, such as aphids, which are the primary insect carrier for potyviruses [54,64,65,66,67]. 

### 3.4. Effect of Nanoclays on Antioxidant Enzymes Activity

**Figure 7:**
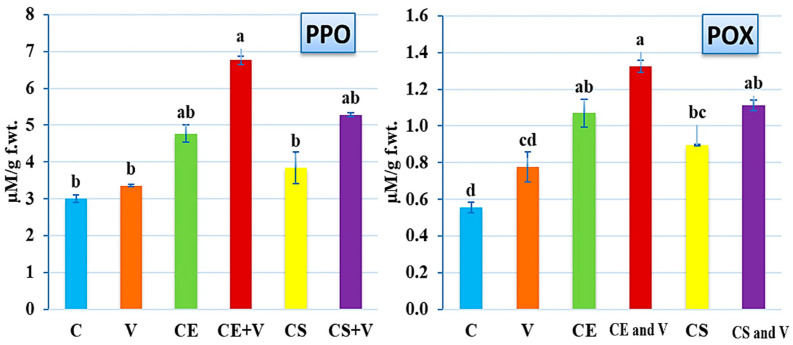
Effect of the foliar application of nanoclay on the PPO and POX of potato plants at 28 days after PVY inoculation. C: control potato plants; V: potato plants inoculated with PVY; CE: potato plants treated with Egyptian nanoclay; CE and V: potato plants inoculated with Egyptian nanoclay 48 h before PVY inoculation; CS: potato plants treated with standard nanoclay; CS and V: potato plants inoculated with standard nanoclay 48 h before PVY inoculation. The columns reflect the average value of five biological replicates, while the bars represent the standard deviation (SD). According to Tukey’s HSD test (*p* ≤ 0.05), the values of each column following the same letter (a/b/c/d/e/f) do not significantly differ.

Because antioxidant enzymes are a key part of a plant’s defense against a wide range of plant pathogens [68], a goal of this study was to compare the activities of PPO and POX in potato plants that were treated with nanoclay and those that were not upon PVY infection (Figure 7). According to the enzyme assay results, the PPO levels significantly increased in the CE and V treatment (6.77 ± 0.12 µM/g f.wt.) compared with the control treatment (3.0 ± 0.11 µM/g f.wt.), as shown in Figure 7. When standard nanoclay was applied to potato plants, the PPO level rose in the CS and V treatment (5.82 ± 0.05 µM/g f.wt.), whereas the CE treatment showed an increase of 4.76 ± 0.23 µM/g f.wt. (Figure 7). There were no discernible differences between the C, V, and CS treatments (Figure 7). These data suggest that nanoclay, particularly of the Egyptian type, plays a function in the up-regulation of the PPO genes in potato plants, which is an important component of the plant’s defense strategy against PVY infection. As a consequence, the overexpression of PPO in a variety of plants has been reported to possess defensive capabilities against bacterial infection [69], fungal infection [70], and viral infection [71] in different plant species. Furthermore, the results showed a significant up-regulation in the POX level upon PVY infection and nanoclay treatments (Figure 7). The highest level of POX (1.32 ± 0.13 µM/g f.wt.) was reported in CE and V treatment plants, followed by CS and V treatment plants and CE treatment plants with 1.11 ± 0.09 µM/g f.wt. and 1.07 ± 0.11 µM/g f.wt., respectively (Figure 7). The virus (V) treatment exhibited a POX level of 0.78 ± 0.06 µM/g f.wt. with no significant change from the control (0.56 ± 0.03 µM/g f.wt.) (Figure 7). The greatest amount of POX was detected in the CE and V treatment, confirming Egyptian nanoclay’s efficiency in increasing POX levels and thereby enhancing tomato plant immunity. To a remarkable extent, POX improves a plant’s resistance to infection by stimulating lignin production based on reactive oxygen species. The physical barrier created by lignin deposition is more effective at preventing viral infection [72]. Our results match those of other studies [52,73] that considered the ability of different nanoparticles to increase the activity of plant enzymes in response to ROS. Additionally, ROS can harm the plant’s membrane lipids, proteins, and DNA, leading to symptom development rather than defense or acclimation if the timing and magnitude of ROS accumulation are not tightly controlled by the plant’s antioxidant system [74,75].

### 3.5. Effect of Nanoclays on the Accumulation Level of PVY-CP 

**Figure 8:**
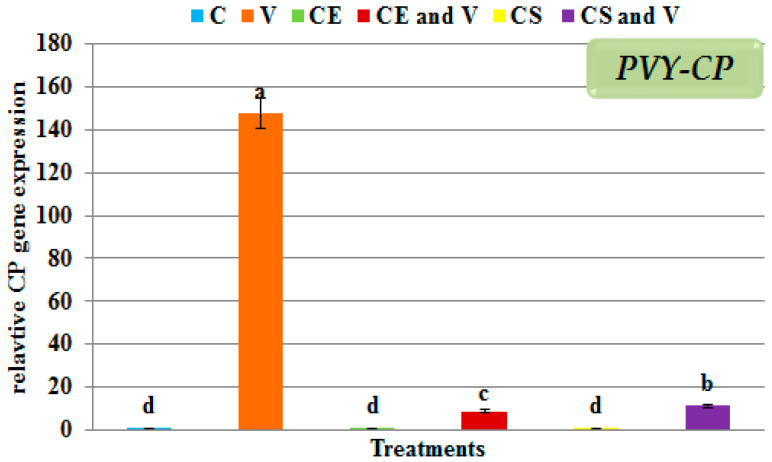
The quantitative gene expression levels of *PVY-CP* in PVY-infected potato leaves at 28 days post-inoculation. C: control potato plants; V: potato plants inoculated with PVY; CE: potato plants treated with Egyptian nanoclay; CE and V: potato plants inoculated with Egyptian nanoclay 48 h before PVY inoculation; CS: potato plants treated with standard nanoclay; CS and V: potato plants inoculated with standard nanoclay 48 h before PVY inoculation. The columns reflect the average value of five biological replicates, while the bars represent the standard deviation (SD). According to Tukey’s HSD test (*p* ≤ 0.05), the values of each column following the same letter (a/b/c/d/e/f) do not significantly differ.

The highest significant accumulation of gene expression of *PVY-CP* (147.65 fold) was only observed in the virus-infected leaves (Figure 8) compared with the controls used in this study (C, CE, and CS treatments). The lowest *PVY-CP* accumulation level was a relative gene expression of 8.86 fold in the potato plants treated with Egyptian nanoclay 48 h before PVY inoculation and a gene expression level of 11.16 fold in the potato plants treated with standard nanoclay 48 h before PVY inoculation (Figure 8). This is the first report on the effect of nanoclay on plant virus accumulation; there have been no studies on the effect of nanoclay on the accumulation of a virus using the qRT-PCR technique.

### 3.6. Transcriptional Expression Levels of Defense-Related Genes 

**Figure 9:**
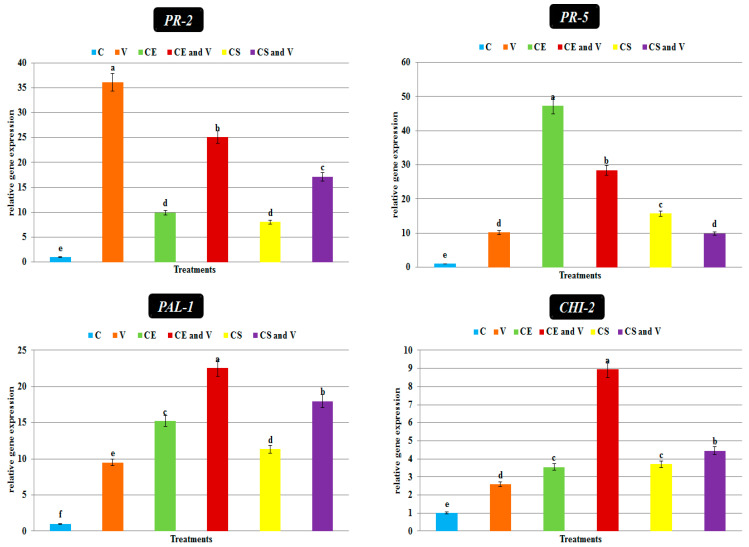
The quantitative gene expression levels of two pathogenesis-related protein genes (*PR-2* and *PR-5*), one polyphenolic gene (*PAL-1*), and one flavonoid gene (*CHI-2*) at 28 dpi. C: control potato plants; V: potato plants inoculated with PVY; CE: potato plants treated with Egyptian nanoclay; CE and V: potato plants inoculated with Egyptian nanoclay 48 h before PVY inoculation; CS: potato plants treated with standard nanoclay; CS and V: potato plants inoculated with standard nanoclay 48 h before PVY inoculation. The columns reflect the average value of five biological replicates, while the bars represent the standard deviation (SD). According to Tukey’s HSD test (*p* ≤ 0.05), the values of each column following the same letter (a/b/c/d/e/f) do not significantly differ.

Generally, antiviral agents work through two mechanisms: the direct and indirect reduction of viral replication via the simultaneous activation of the host’s innate immune system and induction of SAR against viral infection [76,77]. The foliar nanoclay applications induced and activated the transcriptional levels of two pathogenesis-related proteins (*PR-2* and *PR-5*) and two polyphenolic genes (*PAL-1* and *CHI-2*) (Figure 9). *PR-2* encodes β-1, 3-glucanases, which play an important role in the cleavage and hydrolysis of the β-1, 3-glucans, the main component of plant cell walls [78,79]. The results showed that *PR-2* was up-regulated and showed the highest expression level (36.05 fold) in the virus treatment compared with the control (Figure 9). Similarly, Oide et al. [80] showed a clear induction of *PR-2* during viral infections in Arabidopsis. The authors of another study discovered the role of β-1,3-glucanases in cell-to-cell communication viruses in an experiment with a tobacco mutant. Antisense produced a lower level of a class I β-1,3-glucanase transformation. Susceptibility to viral infection is increased in this mutant line. Furthermore, when the coding sequence for β-1,3-glucanases was cloned into TMV, the virus moved more quickly through the cells [81]. Moreover, the substrate for 1,3-glucanase is callose, which is produced in response to viral infection and acts as a physical barrier to virus dissemination. The induction of *PR-2* may diminish callose buildup and facilitate virus multiplication and spread [82]. On the other hand, the treatment of potato plants with nanoclay in our study resulted in decreasing *PR-2* expression (Figure 9). The CE and V treatment and the CS and V treatment exhibited relative expression level changes of 25.10 and 17.12 fold, respectively, higher than the control. Consequently, the foliar application of nanoclay could result in PVY movement limitation between cells by decreasing *PR-2* activity.

In the current study, *PR-5* was up-regulated in all treatments compared with the control. The CE treatment showed the highest transcriptional level (47.27 fold), followed by the CE and V treatment (28.45 fold) and the CS treatment (15.72 fold), compared with the control (Figure 9). The CS and V treatment exhibited a relative expression level of 9.94 fold, with no significant change in virus treatment expression level (10.16 fold). The thaumatin-like protein encoding the *PR-5* gene is localized in cell vacuoles and has antifungal properties [83]. The expression of *PR-5* in *Arabidopsis thaliana* was shown to be elevated following infection with beet severe curly top virus [84]. Moreover, tobacco plants infected with the tobacco vein banding mosaic virus showed an increased expression of the *PR-5* gene [85]. Therefore, the increased transcription and accumulation levels of *PR-5* after the foliar application of nanoclay could play a significant role in plant defense against pathogen attacks. The significant up-regulation of expression of *PAL* was observed in all treatments compared with the control (Figure 9) in our study. Compared with the control potato plants, the highest gene expression (22.55 fold) was shown in the CE and V treatment (Figure 9), followed by the CS and V treatment, CE treatment, CS treatment, and V treatment, with relative expression levels of 17.99 fold, 15.25 fold, 11.32 fold, and 9.5 fold, respectively (Figure 9). Regarding the *CHI-2* transcript, the CE and V treatment and the CS and V treatment exhibited the highest accumulation levels, with transcriptional levels of 8.94 fold and 4.45 fold, respectively, higher than the control (Figure 9). No significant change was reported between the CE and CS treatments. The PVY (V) treatment showed a transcriptional level of 2.6-fold higher than the control (Figure 9). It is well-known that plants use the *PAL* gene to respond to biotic and abiotic stress. The phenylpropanoid pathway plays a significant role in plant defense systems, providing structural and chemical barriers to pathogen invasion resistance. Phenylpropanoid pathway genes were previously shown to overexpressed after pathogen infection, resulting in increased enzymatic activity and phenolic compound accumulation [86,87]. Similarly, the phenylpropanoid pathway gene *PAL1* plays a significant role in developing cassava brown streak virus resistance in cassava plants. Its early induction is crucial for CBSV resistance [88]. These elevated genes’ transcriptional expression suggests that they play a protective role against ToMV. The pathogen-dependent accumulation of flavonoids and isoflavonoid phytoalexins is regulated by the *CHI-2* encoded gene, which has a protective role in plant immunity. The up-regulation of *CHI-2* gene expression in mycorrhizal colonization induces a plant immune system against ToMV-infected plants [38].

## 4. Conclusions

This study is first report of the antiviral activity of nanoclay against PVY. The results of a nanoclay structural analysis were confirmed with FTIR, TEM, SEM, and EDS. The foliar treatment of potato plants with 1% nanoclay improved potato development, reduced disease symptoms, and decreased PVY accumulation levels relative to untreated plants. There were also significant increases in antioxidant enzyme levels (PPO and POX) and decreases in oxidative stress indicators (MDA and H_2_O_2_). In addition, a considerable increase in the transcriptional levels of defense-related genes (*PAL-1*, *PR-5*, and *CHI-2*) was detected. Based on the collected data, using nanoclay to fight PVY could be a good idea and a safe, low-cost, and environmentally friendly material.

## Figures and Tables

**Table 1:** Nucleotide sequences of qPCR primers used for defense response in this study.

Gene	Direction	Primer Sequence (5′–3′)	Functional Annotation	Related Pathway
*PR-2*	Forward	TATAGCCGTTGGAAACGAAG	β-1,3-glucanases	Pathogenesis-related proteins
	Reverse	CAACTTGCCATCACATTCTG		
*PR-5*	Forward	ACCTCTTCCGCTGTCCTC	Thaumatin-like protein	Pathogenesis-related proteins
	Reverse	GAAGACGACTTGGTAGTTGC		
*PAL-1*	Forward	ACGGGTTGCCATCTAATCTGACA	Phenylalanine ammonia-lyase	Phenylpropanoid biosynthetic
	Reverse	CGAGCAATAAGAAGCCATCGCAAT		
*CHI-2*	Forward	GGCAGGCCATTGAAAAGTTCC	Chalcone isomerase 2	Flavonoid/isoflavonoid biosynthesis
	Reverse	CTAATCGTCAATGATCCAAGCGG		
*B-actin*	Forward	ATGCCATTCTCCGTCTTGACTTG	βeta-actin	Housekeeping
	Reverse	GAGTTGTATGTAGTCTCGTGGATT		
*PVY-CP*	Forward	CAACTCCAGATGGAACAATTG	*Potato Virus Y*-coat protein	Virus replication
	Reverse	CCATTCATCACAGTTGGC		

**Table 2:** FTIR spectra list of band positions for Egyptian and standard nanoclay in this study.

Weave Number (cm^−1^)	Functional Group	
	Egyptian Nanoclay	Standard Nanoclay
432.10		Si-O-Si bending
509.46	Fe-O Fe2O3 Si-O-Al stretching	
627.50		Si-O-Si of quartz
682.05	Si-O-Si bending	
786.03	Si-O quartz	
913.41	C=C binding alkane	
978.82		OH deformation, linked to 2Al^2^
988.67	OH deformation, linked to 2Al^2^	
1191.57		Al-O as Si cage (TO^4^)
1463.36	C-H stretching	
1632.73	H-O-H deformation of water	
1642.22		H-O-H deformation of water
2327.72		O=C=O Carbon dioxide
3348.83		H-O-H stretching, Absorbed water
3611.60	OH stretching of inner-surface hydroxyl groups	
3405.15	OH of water	

**Table 3:** EDS analysis of Egyptian nanoclay and standard nanoclay.

Elements	Egyptian Nanoclay			Standard Nanoclay		
	Intensity	Weight %	Atomic %	Intensity	Weight %	Atomic %
**C**	722	47.34	63.67	698	31.43	45.58
**O**	225	21.42	21.63	292	31.16	33.93
**Na**	96	1.21	0.85			
**Mg**	74	0.79	0.52	134	5.15	3.69
**Al**	136	5.29	3.16	134	2.42	1.56
**Si**	187	12.09	6.95	560	19.61	12.16
**S**				65	0.64	0.35
**K**	43	0.31	0.13	42	0.18	0.08
**Ti**	41	0.38	0.13	38	0.16	0.06
**Fe**	82	2.89	0.84	112	4.95	1.54
**Ni**				40	0.16	0.05
**Co**	44	0.20	0.05			
**Cu**	118	8.09	2.06	88	3.34	0.92
**Ta**				94	0.80	0.08

**Table 4:** Effect of Egypt and standard nanoclays on the growth parameters of potato plants infected with PVY (28 days after inoculation). C: control potato plants; V: potato plants inoculated with PVY; CE: potato plants treated with Egyptian nanoclay; CE and V: potato plants inoculated with Egyptian nanoclay 48 h before PVY inoculation; CS: potato plants treated with standard nanoclay; CS and V: potato plants inoculated with standard nanoclay 48 h before PVY inoculation.

Treatment *	Plant Height (cm)	Shoot Length (cm)	Root Length (cm)	Shoot Fresh Weight (g)	Root Fresh Weight (g)
**C**	24.33 ± 1.53 c	15.50 ± 1.8 bc	07.66 ± 1.53 c	3.53 ± 0.50 b	0.99 ± 0.18 ab
**V**	14.50 ± 1.32 d	09.93 ± 1.04 d	05.33 ± 1.52 c	1.57 ± 0.60 c	0.50 ± 0.20 b
**CE**	37.66 ± 2.08 a	15.00 ± 1.0b c	22.66 ± 2.52 a	5.27 ± 0.55 a	0.93 ± 0.21 ab
**CE and V**	30.33 ± 2.51 b	16.33 ± 2.08 b	13.66 ± 1.53 b	3.77 ± 1.05 b	0.78 ± 0.18 ab
**CS**	35.66 ± 2.08 a	20.50 ± 1.32 a	15.17 ± 1.04 b	4.67 ± 1.08 ab	1.16 ± 0.56 a
**CS and V**	29.83 ± 1.61 b	13.67 ± 0.58 c	16.66 ± 3.05 b	2.23 ± 0.35 c	0.81 ± 0.15 ab

* According to Tukey’s HSD test, the values of each column following the same letter are not significantly different at *p* ≤ 0.05. Each value reflects the average value of five biological replicates with its standard deviation (±SD).

## Data Availability

Not applicable.
